# Supplementary material for: High oxygen barrier packaging materials from protein-rich single-celled organisms
Source: Commun Chem. 2025 Oct 6;8:297. doi: 10.1038/s42004-025-01720-x (PMC12500970; doi:10.1038/s42004-025-01720-x)
Supplement: Supplementary file 2 — Supporting Information [file 42004_2025_1720_MOESM2_ESM.pdf]

Supporting Information

High Oxygen Barrier Packaging Materials from Protein-rich Single-Celled Organisms

Kiran Reddy Baddigam[1]**, *Bor Shin Chee*[2], *Elodie Guilloud* [1], *Chaitra Venkatesh*[2], *Helena Koninckx*[3], *Kim Windey*[4], *Margaret Brennan Fournet*[2], *Mikael Hedenqvist*[1]**, *Anna J. Svagan*[1]**

[1]KTH Royal Institute of Technology, Dept. of Fibre and Polymer Technology, SE-100 44 Stockholm, Sweden

[2]TUS Technological University of the Shannon – Midlands Midwest, Centre for Polymer Sustainability, PRISM Research Institute, Athlone, Ireland

[3]Avecom nv, 9032 Wondelgem, Ghent, Belgium

[4]Valpromic nv, 9032 Wondelgem, Ghent, Belgium

Supplementary Table 1. Amino acid composition of the microbial protein and lipid composition.

**Amino acids**	**(g/kg)**	**(%-amino)**
Cysteine	5.4	0.9 %
Methionine	11.6	2.0 %
Valine	41.9	7.2 %
Isoleucine	28.2	4.9 %
Leucine	47.4	8.2 %
Tyrosine	23.3	4.0 %
Phenylalanine	27.3	4.7 %
Lysine	32.6	5.6 %
Histidine	11.0	1.9 %
Arginine	39.0	6.7 %
Aspartic Acid + Asparagine	56.7	9.8 %
Threonine	30.9	5.3 %
Serine	23.3	4.0 %
Glutamic acid + Glutamine	76.8	13.2 %
Proline	28.4	4.9 %
Glycine	37.1	6.4 %
Alanine	50.5	8.7 %
Tryptophan	9.8	1.7 %
**Lipids**	**(%-fat)**	
Neutral Lipid (%-fat)	26.0 %	
Glycolipids (%-fat)	16.2 %	
Phospholipids	57.8 %	

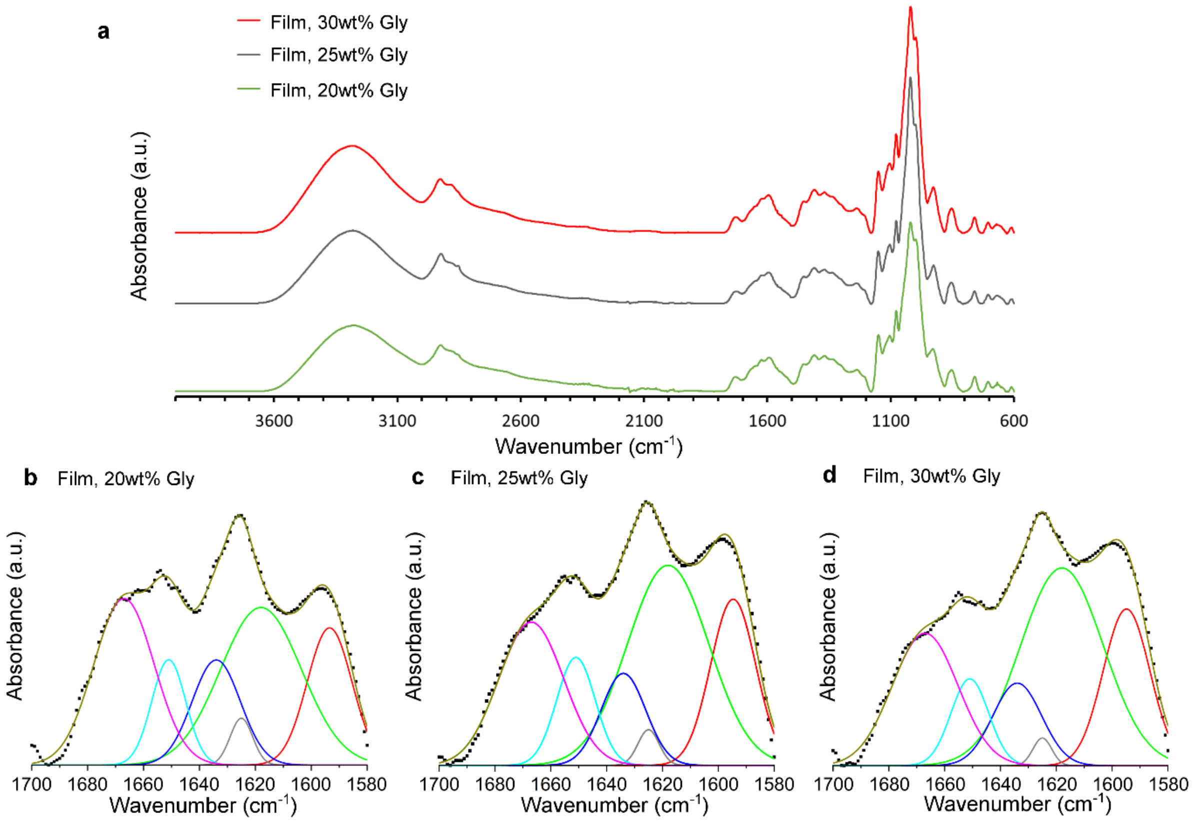

Supplementary Figure 1. FTIR spectra for (a) films with increasing amounts of glycerol (20 wt%, 25 wt% and 30 wt%). In (b-c): deconvoluted FTIR spectra in the amide I region 1580 – 1700 cm^{-1}. The dotted lines are experimental base-line corrected FTIR data.

Supplementary Table 2. Secondary structures of the proteins present in the MB films with different glycerol contents. The values within parenthesis are the uncertainties of the optimized values.

	Secondary structure	MB Film		
	Glycerol content (wt%)	20	25	30
**Position (cm^{-1})**	**Assignment**			
1680	β-Sheets, weakly hydrogen-bonded peptide groups	n.a.	n.a.	n.a.
1667	β-Turns	30.9 (0.4)	26.8 (0.5)	26.4 (0.5)
1658	α-Helices	n.a.	n.a.	n.a.
1651	α-Helices and random coils	11.0 (1.1)	12.1 (1.9)	10.0 (1.8)
1644	Unordered	n.a.	n.a.	n.a.
1634	β-Sheets, weakly hydrogen-bonded peptide groups	15.6 (5.6)	11.8 (8.6)	12.2 (8.9)
1625	β- Sheets, strongly hydrogen-bonded peptide groups	3.2 (1.2)	2.2 (1.4)	1.6 (1.0)
1618	β- Sheets, strongly hydrogen-bonded peptide groups	39.3 (10.7)	47.1 (17.6)	49.8 (18.4)
	**Secondary structures summarized**	(%)	(%)	(%)
	β-Turns	30.9	26.8	26.4
	α-Helices and random coils/unordered	11.0	12.1	10.0
	β-Sheets, weakly hydrogen-bonded peptide groups	15.6	11.8	12.2
	β- Sheets, strongly hydrogen-bonded peptide groups	42.5	49.3	51.4

n.a. = not attained.

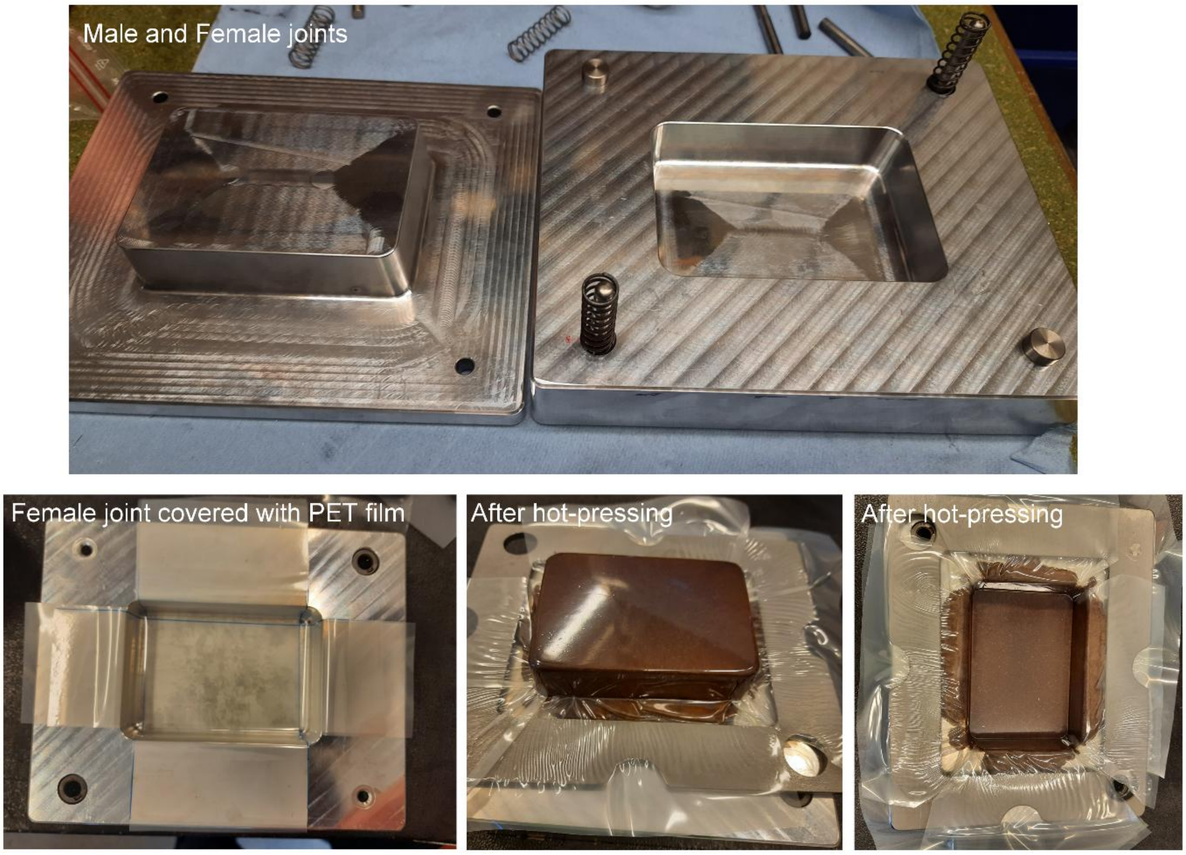

Supplementary Figure 2. Picture of the steel mold parts used for producing the MB tray. The joints were covered with PET film prior to hot-pressing.
