# Supplementary material for: High oxygen barrier packaging materials from protein-rich single-celled organisms
Source: Commun Chem. 2025 Oct 6;8:297. doi: 10.1038/s42004-025-01720-x (PMC12500970; doi:10.1038/s42004-025-01720-x)
Supplement: Supplementary file 3 — Description of Additional Supplementary Files [file 42004_2025_1720_MOESM3_ESM.pdf]

Description of Additional Supplementary Files

File name: Supplementary Data 1

Description: Numerical source data for graphs (FTIR, Mechanical and OP data)

File name: Supplementary Movie 1

Description: Demonstration of a microbial biomass-based film.

File name: Supplementary Movie 2

Description: Demonstration of a microbial biomass-based tray.
